# Hydrogen Sulfide: An Emerging Regulator of Oxidative Stress and Cellular Homeostasis—A Comprehensive One-Year Review

**DOI:** 10.3390/antiox12091737

**Published:** 2023-09-07

**Authors:** Constantin Munteanu, Marius Alexandru Turnea, Mariana Rotariu

**Affiliations:** 1 Teaching Emergency Hospital “Bagdasar-Arseni” (TEHBA), 041915 Bucharest, Romania; 2 Faculty of Medical Bioengineering, University of Medicine and Pharmacy “Grigore T. Popa” Iași, 700454 Iași, Romania; mariana.rotariu@umfiasi.ro

**Keywords:** hydrogen sulfide, oxidative stress, cellular homeostasis, biochemical mechanisms, therapeutic potential of H_2_S

## Abstract

Hydrogen sulfide (H_2_S), traditionally recognized as a toxic gas, has emerged as a critical regulator in many biological processes, including oxidative stress and cellular homeostasis. This review presents an exhaustive overview of the current understanding of H_2_S and its multifaceted role in mammalian cellular functioning and oxidative stress management. We delve into the biological sources and function of H_2_S, mechanisms underlying oxidative stress and cellular homeostasis, and the intricate relationships between these processes. We explore evidence from recent experimental and clinical studies, unraveling the intricate biochemical and molecular mechanisms dictating H_2_S’s roles in modulating oxidative stress responses and maintaining cellular homeostasis. The clinical implications and therapeutic potential of H_2_S in conditions characterized by oxidative stress dysregulation and disrupted homeostasis are discussed, highlighting the emerging significance of H_2_S in health and disease. Finally, this review underscores current challenges, controversies, and future directions in the field, emphasizing the need for further research to harness H_2_S’s potential as a therapeutic agent for diseases associated with oxidative stress and homeostatic imbalance. Through this review, we aim to emphasize H_2_S’s pivotal role in cellular function, encouraging further exploration into this burgeoning area of research.

## 1. Introduction

A captivating theme in modern biology is the ability of simple molecules to orchestrate complex physiological functions [1]. Hydrogen sulfide (H_2_S) [2], historically notorious merely as a hazardous, colorless, flammable gas with a characteristic rotten egg odor, is now understood to be a biological signaling molecule [3]. This shift in perception is due to the recognition of H_2_S as a significant player in diverse physiological and pathological processes [4]. This small molecule, considered as the third gasotransmitter alongside nitric oxide (NO) and carbon monoxide (CO), exerts a plethora of effects in mammalian physiology, such as vasodilation [5], vascular tone [6], modulating the inflammatory response [7,8], neurotransmission [9], antioxidant properties [10], apoptosis [11] cellular survival [12], regulating cellular metabolism [13], or acting as a cytoprotectant [14]. As a multifaceted molecule [15,16] with profound biological implications, H_2_S has increasingly been at the center of scientific scrutiny. This review aims to shed light on the emerging role of H_2_S in regulating oxidative stress and maintaining cellular homeostasis, which is crucial for maintaining proper cellular functioning and ensuring the organism’s survival [17].

It is essential to acknowledge that the intricate biological effects attributed to H_2_S are not directly induced by the gas itself. Rather, H_2_S triggers a complex array of oxidative modifications, prominently including persulfidation, disulfide formation, and polysulfide generation. These oxidative transformations operate as fundamental modulators of H_2_S’s versatile biological functions. The induction of such oxidative modifications is a prerequisite for H_2_S to proficiently engage in the regulation of cellular processes. Through these chemical transformations, H_2_S engages in selective interactions with specific biomolecules, including proteins, redox-sensitive molecules, and signaling entities. This interplay initiates an expansive spectrum of physiological responses. Consequently, the intricate and multifaceted biological ramifications of H_2_S emanate from its nuanced interplay with cellular components, facilitated by these oxidative modifications [18].

H_2_S is synthesized endogenously in mammalian tissues by the enzymatic degradation of sulfur-containing amino acids, primarily cysteine. Three primary enzymes are involved in this process, each differing in distribution, regulation, and function. These are cystathionine beta-synthase (CBS), cystathionine gamma-lyase (CSE), and 3-mercaptopyruvate sulfurtransferase (3-MST). These enzymes are strategically distributed in various mammalian tissues, emphasizing the widespread influence of H_2_S [19,20,21,22].

Cystathionine β-Synthase (CBS), a pyridoxal 5′-phosphate (PLP)-dependent enzyme, is primarily localized within the brain and central nervous system [23]. This enzyme takes center stage in the transsulfuration pathway, an essential biochemical pathway in the human body involving the interconversion of sulfur-containing amino acids, methionine, and cysteine [24]. CBS initiates the transsulfuration pathway by catalyzing its first step [25,26,27,28].

Cystathionine γ-Lyase (CSE), another PLP-dependent enzyme predominantly expressed in peripheral tissues, particularly in the liver, kidney, vascular smooth muscle, and endothelial cells, is a pyridoxal phosphate-dependent enzyme that catalyzes the final step in the transsulfuration pathway in the endogen production of H_2_S [29,30,31,32].

The 3-Mercaptopyruvate Sulfurtransferase (3-MST), in conjunction with cysteine aminotransferase (CAT), 3-MST, also produces H_2_S. The CAT/3-MST pathway is a significant source of H_2_S in the brain and vascular endothelial cells [33].

Many published articles cover the aspects related to H_2_S biosynthesis, enzymes involved, places of synthesis, and their roles in various body organs. Summarizing cutting-edge research from the past year, a novel pathway in mammals involves peroxisome-dependent DAO converting D-cysteine into 3MP, leading to H_2_S production in mitochondria. Additionally, H_2_S can be stored as bound sulfane sulfur in specific cysteine residues of target proteins, suggesting a role as a signaling molecule in the nervous system [34].

The biological functions of H_2_S are versatile and multidirectional, playing pivotal roles in vasodilation, neurotransmission, inflammation modulation, oxidative balance, cellular apoptosis, angiogenesis [35], glucose regulation, energy metabolism [36], and cellular survival and longevity [37]. But, all these biological functions are also part of cellular homeostasis [38,39].

H_2_S exhibits diverse physiological roles, acting as a potent vasodilator by activating ATP-sensitive potassium channels, leading to smooth muscle relaxation and consequent decreased blood pressure. Moreover, it promotes angiogenesis through upregulating VEGF and VEGFR-2, influencing wound healing, inflammation, and tumor growth [35,40].

In the central nervous system, H_2_S functions as a neuromodulator, modulating NMDA receptor responses and facilitating memory formation through hippocampal long-term potentiation. It also exerts anti-inflammatory effects by inhibiting NF-κB activation. Additionally, H_2_S displays cytoprotective effects by scavenging ROS and enhancing antioxidant enzyme activities, mitigating oxidative stress and cellular damage [10,41].

H_2_S is also involved in the regulation of glucose regulation and energy metabolism [13]. It enhances glucose uptake in peripheral tissues, contributing to overall glucose homeostasis. Furthermore, H_2_S plays a crucial role in mitochondrial function, influencing ATP synthesis and regulating metabolic energy production [42]. It modulates the activity of critical enzymes in glycolysis [43], gluconeogenesis, and the citric acid cycle, underscoring its role in maintaining metabolic homeostasis [44,45,46].

Oxidative stress denotes a physiological state characterized by an imbalance between the production of reactive oxygen species (ROS) and the system’s ability to neutralize these highly reactive molecules or repair the damage caused by them [47]. ROS are generated as natural by-products of oxygen metabolism and play a critical role in cell signaling and homeostasis [48]. However, when faced with environmental stress, ROS levels can drastically rise, resulting in significant damage to cell structures—a phenomenon known as oxidative stress [49]. Oxidative stress manifests as a cascade of destructive events affecting cellular proteins, lipids, and nucleic acids, impairing cellular functions and leading to various pathological conditions, including but not limited to cardiovascular disorders, neurodegenerative diseases, cancer, and diabetes [50].

The concentration-dependent dichotomy of H_2_S in cellular survival and apoptosis is concentration-dependent. Physiological levels of H_2_S confer cytoprotection, inhibiting cellular apoptosis or enhancing cellular resistance to oxidative stress, contributing to cellular longevity [51]. Conversely, H_2_S may induce apoptosis at higher concentrations, particularly in cancer cells, by impacting mitochondrial function [52]. H_2_S can cause the production of glutathione, a powerful antioxidant, thereby bolstering the cellular antioxidant defense system [53]. Furthermore, it can activate cellular stress response mechanisms, including the Nrf2 pathway, which plays a critical role in the protective mechanism against the harmful effects of oxidative stress [54,55,56].

Cellular homeostasis, on the other hand, encompasses the vast array of processes and mechanisms that cells employ to maintain the stability of their internal environment, even when faced with external changes [57]. It precisely regulates critical factors, such as pH, ion concentrations, temperature, nutrient supply, waste removal, and redox balance [58]. Disrupting any of these parameters can destabilize cellular homeostasis, contributing to cellular dysfunction, the initiation of pathological processes, and disease development [59,60].

**Figure 1:**
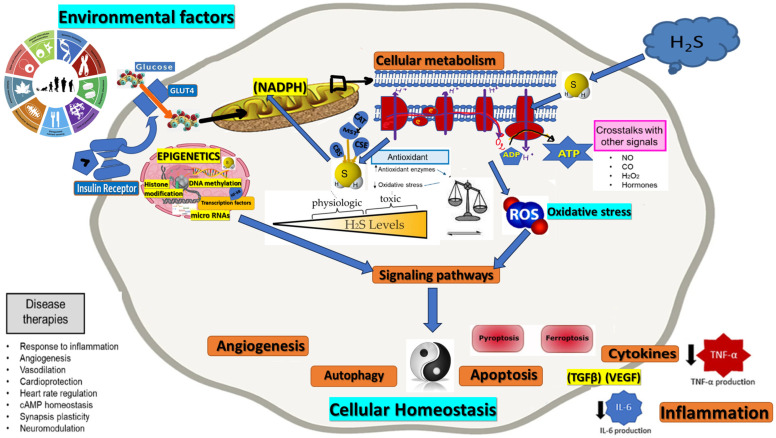
Role of H_2_S in oxidative stress and cellular homeostasis intersection. Consequences regarding various physiological functions, including angiogenesis, autophagy, apoptosis, inflammation—cytokines level reduction (black arrows).

With oxidative stress and cellular homeostasis playing central roles in cellular function and disease pathology, the intersection of these processes with H_2_S bioavailability and metabolism presents a fascinating study area [61]. This review comprehensively provides an overview of the complex relationship between H_2_S, oxidative stress, and cellular homeostasis. We aim to delve into the current understanding of the underlying biochemical and molecular mechanisms that dictate the role of H_2_S in these processes, drawing upon the latest research from in vitro, in vivo, and clinical studies (Figure 1).

While previous reviews have discussed H_2_S’s involvement in vasodilation, neurotransmission, inflammation modulation, oxidative balance, apoptosis, angiogenesis, glucose regulation, energy metabolism, cellular survival, and longevity [35,36,37], our review uniquely ties these biological functions to the overarching concept of cellular homeostasis [38,39]. We explore further how H_2_S orchestrates multifaceted responses to maintain equilibrium within the cell, offering readers a comprehensive view of its role in various physiological processes.

In essence, the biological roles of H_2_S are integral to maintaining the complex orchestration of various physiological processes [62]. Any imbalance in its endogenous production or metabolism can lead to pathological conditions. This highlights the importance of ongoing research into the intricate biochemistry of H_2_S and its physiological and pathological roles in health and disease. As a regulator of oxidative stress and cellular homeostasis, H_2_S holds immense therapeutic potential for various conditions [41].

## 2. Methods

Recognizing the vast volume of the literature on H_2_S, we have conducted a systematic review, meticulously selecting and analyzing a subset of the most relevant and impactful articles. This allows us to distill key findings, present critical analyses, and offer insightful perspectives to the readers.

**Figure 2:**
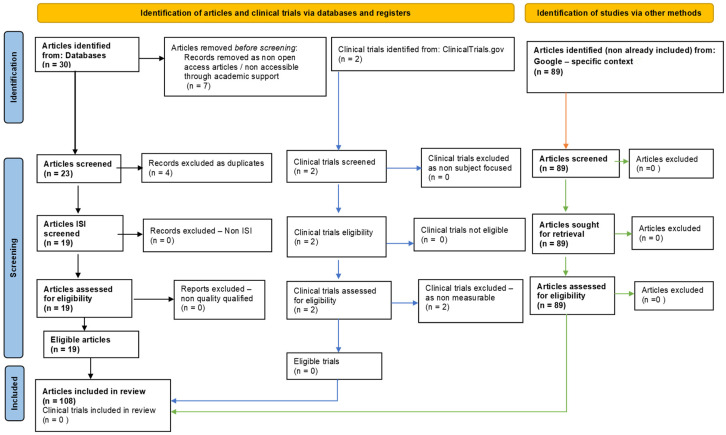
Adapted PRISMA flow diagram, customized for our study.

This one-year systematic review (23 July 2022–23 July 2023) followed PRISMA guidelines and is registered on PROSPERO, ID: 453191. To ensure comprehensive coverage of the relevant literature, we searched multiple databases, including PubMed, Scopus/Elsevier, Web of Science, and Google Search. The search strategy involved using specific keywords and medical subject headings (MeSH) related to hydrogen sulfide, oxidative stress; cellular homeostasis; and biochemical mechanisms. After removing non-eligible and duplicate references, our review included 108 relevant studies (Figure 2).

The criteria for selecting articles were determined based on their alignment with our review’s focus, i.e., the impact of H_2_S on oxidative stress modulation and cellular homeostasis. Inclusion criteria encompassed studies that directly investigated H_2_S’s roles within these contexts, involving both in vitro and in vivo experimental approaches. We also considered clinical studies with implications for translational applications. Exclusion criteria were applied to studies that were not directly relevant to our focus, including those that solely discussed the general biochemistry of H_2_S without delving into its roles in oxidative stress or cellular homeostasis.

To minimize bias, we employed a multistep screening process, involving initial title and abstract screening, followed by full-text assessment, performed independently by all the three authors of this article. Any discrepancies were resolved through consensus discussion. Additionally, to address potential bias, we assessed the diversity of study designs, methodologies, and outcome measures across the selected articles. Our aim was to ensure a balanced representation of different perspectives within the literature and to avoid undue emphasis on a particular subset of studies.

The initial search, performed by the first author in the International Databases, identified a total of 30 articles. After eliminating duplicates, the remaining 19 full-text articles of the selected records were subsequently evaluated to determine their eligibility based on predefined inclusion and exclusion criteria. The 19 articles were analyzed to assess their relevance to our research question by all the authors, and the analysis is included in Tables 1 and 2, based on their link to oxidative stress (Table 2) or to homeostasis (Table 2).

Additionally, to support the ideas expressed in this review, bibliographical references were identified by other methods—Google contextual search, restricted to last-year time.

The selection of predominantly review articles in this systematic review was influenced by the specific focus on summarizing and analyzing recent advancements in the understanding of hydrogen sulfide’s role in oxidative stress and cellular homeostasis.

During the search process, we did encounter a limited number of original research papers directly addressing the role of hydrogen sulfide in oxidative stress and cellular homeostasis. However, these original research papers often focused on specific aspects of the topic, such as molecular mechanisms or specific pathways, rather than providing a comprehensive overview of the entire field. Additionally, review articles often incorporated insights from multiple original research studies, enhancing the synthesis of information. While original research papers provided invaluable insights, the review articles offered a consolidated perspective by summarizing and critically evaluating findings from various studies, including both original research and experimental work.

It is worth noting that our focus on recent advancements within the past year further contributed to the prominence of review articles, as they are more likely to provide up-to-date summaries of the latest research findings. While we acknowledge the significance of original research papers, this systematic review aims to offer a comprehensive understanding of the current landscape through the lens of very recent data.

## 3. Results

### 3.1. Hydrogen Sulfide and Oxidative Stress—Last-Year Data

The intricate relationship between hydrogen sulfide (H_2_S) and oxidative stress is an area of extensive interest in molecular biology, biochemistry, and medicine [63]. Oxidative stress, characterized by an imbalance between the generation of reactive oxygen species (ROS) and the ability of the biological system to detoxify these reactive intermediates, has significant implications for cellular function, tissue integrity, and the pathogenesis of numerous diseases [64,65].

Emerging as the third endogenous gaseous signaling molecule or ‘gasotransmitter’ following nitric oxide (NO) and carbon monoxide (CO), H_2_S has attracted substantial attention due to its potential role in mitigating oxidative stress. Uniquely, H_2_S displays both direct and indirect antioxidant capacities, a bifunctional characteristic that underscores its critical position within the antioxidant defense machinery [66,67,68].

H_2_S’s direct antioxidant capacity is attributable to its chemical properties, which allow it to neutralize ROS. For example, H_2_S can react with superoxide anions (O^2−^) to produce a hydrosulfide anion (HS^−^) and hydrogen peroxide (H_2_O_2_). This reaction reduces the concentration of O^2−^, a potent ROS [69,70]. Furthermore, H_2_S can also directly neutralize H_2_O_2_, forming water (H_2_O) and elemental sulfur, disarming the potential harm these reactive species pose [71].

Beyond its role as a direct ROS scavenger, H_2_S also displays indirect antioxidant properties, bolstering the body’s endogenous antioxidant defense systems, achieved by upregulating key antioxidant enzymes, including superoxide dismutase (SOD), catalase, and glutathione peroxidase (GPx) [72,73]. These enzymes, pivotal for maintaining redox homeostasis, can neutralize ROS, thus limiting oxidative damage [74]. Through signaling mechanisms not yet fully elucidated, H_2_S has been demonstrated to induce the expression of these enzymes, thereby enhancing the cellular capacity to counteract ROS and reduce oxidative stress [75].

H_2_S’s protective influence extends to the cellular mitochondria, critical organelles for energy production and cellular metabolism [76]. Under physiological conditions, H_2_S can support mitochondrial function and cellular energy metabolism by participating in electron transport and ATP synthesis [77]. Conversely, under conditions of oxidative stress, H_2_S adopts a cytoprotective role. It attenuates mitochondrial ROS production, promotes mitochondrial biogenesis, and blocks the opening of mitochondrial permeability transition pores (mPTP). Under oxidative insults, these actions significantly preserve mitochondrial integrity, function, and cell survival [78,79].

The interplay between H_2_S and other gasotransmitters, such as NO, also impacts oxidative stress. H_2_S can react with NO to form a new compound, nitroxyl (HNO). Interestingly, HNO exhibits potent antioxidant activity and may contribute to cellular antioxidant defense. This suggests that the interaction between H_2_S and NO may represent an additional mechanism through which H_2_S exerts its antioxidant effects [80].

Despite these seemingly beneficial effects, the impact of H_2_S on oxidative stress is not unequivocally protective. Indeed, H_2_S’s outcomes are highly concentration-dependent [81,82]. Physiological concentrations of H_2_S are generally associated with cytoprotection against oxidative stress [83]. However, elevated H_2_S levels can contribute to oxidative stress, especially under pathophysiological conditions [10]. This paradoxical effect is primarily due to the ability of high concentrations of H_2_S to inhibit cytochrome c oxidase, a specific enzyme in the mitochondrial electron transport chain. Inhibition of this enzyme impedes electron transport, which can culminate in elevated ROS production and exacerbated oxidative stress [84].

This dichotomous role of H_2_S in oxidative stress is reminiscent of the ‘Janus-faced’ characteristic many biological molecules exhibit [85]. The context-dependent nature of H_2_S’s biological actions adds complexity to our understanding of this gasotransmitter’s role in health and disease. A comprehensive exploration of the nuances in H_2_S signaling and identifying the precise tipping point where H_2_S transitions from a cytoprotectant to a cytotoxic agent is essential to fully realize the therapeutic potential of H_2_S in oxidative stress-associated conditions. Advancing research technologies unravel the mysteries surrounding H_2_S and its interplay with oxidative stress (Table 1).

**Table 1:** Synthetic Data on Hydrogen Sulfide and Oxidative Stress.

Ref.	Title	Synthetic Data on Hydrogen Sulfide and Oxidative Stress	Outcomes
[8]	Effect of Hydrogen Sulfide on Essential Functions of Polymorphonuclear Leukocytes	Distinct from information available until 2022, this article emphasizes the dual nature of H_2_S in inflammation, acting both as a pro-inflammatory and anti-inflammatory molecule. It also elaborates on the complex interactions of H_2_S with various signaling pathways, its effects on different organ systems, and its potential therapeutic applications. The detailed exploration of H_2_S’s role in renal disease, including its interaction with uremic toxins and its impact on oxidative stress, offers a novel perspective that contributes to the understanding of H_2_S’s function in human health and disease.	The article elucidates the dual role of hydrogen sulfide (H_2_S) in inflammation, its complex interactions with signaling pathways, and its potential therapeutic applications, particularly in renal disease.
[10]	Advances of H_2_S in Regulating Neurodegenerative Diseases by Preserving Mitochondria Function	A comprehensive insight into the multifaceted role of H_2_S in neurodegenerative diseases, emphasizing its neuroprotective properties. It delves into the specific mechanisms through which H_2_S modulates mitochondrial activity, produces reactive sulfur species, and modifies proteins through sulfhydration. The article emphasizes the potential therapeutic applications of H_2_S in regulating neurodegenerative diseases through anti-oxidative, anti-inflammatory, anti-apoptotic, and S-sulfhydration.	The article reveals hydrogen sulfide’s dual role as a neuromodulator and neuroprotectant, offering new therapeutic avenues in neurodegenerative diseases.
[14]	SOD1 is an essential H_2_S detoxifying enzyme	Contrary to the prevailing knowledge until 2022, the paper uncovers the role of superoxide dismutase [Cu-Zn] (SOD1) as an efficient H_2_S oxidase, essential in limiting cytotoxicity from both endogenous and exogenous sulfide. It highlights SOD1’s ability to convert H_2_S to sulfate under limiting sulfide conditions rapidly and its role in forming per- and polysulfides, which induce cellular thiol oxidation.	The article reveals SOD1’s role as an H_2_S oxidase, essential in limiting H_2_S cytotoxicity, and regulating reactive sulfur species, enriching our understanding of H_2_S detoxification.
[43]	Generation and Physiology of Hydrogen Sulfide and Reactive Sulfur Species in Bacteria	The article presents a nuanced understanding of hydrogen sulfide’s (H_2_S) role in the oxidative stress response, highlighting its combinatorial redox action with hydrogen peroxide (H_2_O_2_) in mediating cytotoxicity and its contrasting protective effect against ROS-mediated killing. This complex interplay, including the formation of sulfheme iron complexes and the impact on catalases, adds to the existing knowledge by elucidating the multifaceted roles of H_2_S in oxidative stress and immunometabolism.	The article reveals hydrogen sulfide’s dual role in oxidative stress, mediating cytotoxicity and offering protection against ROS-mediated killing, enhancing understanding of immunometabolism.
[86]	Hydrogen Sulfide: A Gaseous Mediator and Its Key Role in Programmed Cell Death, Oxidative Stress, Inflammation, and Pulmonary Disease	The article advances the understanding of hydrogen sulfide (H_2_S) in oxidative stress, emphasizing its dual role as an antioxidant and a pro-oxidant. It highlights the complex mechanisms of H_2_S in quenching free radicals and its potential therapeutic targeting in pulmonary diseases, adding nuance to existing knowledge.	Insights into the tightly controlled metabolism of H_2_S in mammals, achieved through physiological enzymes catalyzed reactions.
[87]	Hydrogen sulfide: A new therapeutic target in vascular diseases	Emphasizes the intricate relationship between H_2_S and oxidative stress in regulating blood pressure. It details the mechanisms by which H_2_S acts as a vasorelaxant agent, its interaction with nitric oxide (NO), and the effects of various H_2_S donors in treating HBP. The potential of H_2_S as a therapeutic target for hypertension, including its role in inhibiting inflammation, suppressing vascular smooth muscle cell proliferation, and mitigating oxidative stress, thereby contributing to the understanding of H_2_S’s multifaceted role in cardiovascular health.	The article reveals hydrogen sulfide’s multifaceted role in regulating hypertension, emphasizing its potential as a therapeutic target in cardiovascular health.
[88]	Hydrogen Sulphide-Based Therapeutics for Neurological Conditions: Perspectives and Challenges	A comprehensive insight into the catabolism of H_2_S and its role in oxidative stress within the brain. Specifically, it highlights the interplay between enzymes, like sulfide quinone oxidoreductase (SQR) and its homolog SQRDL, along with neuroglobin, in the metabolism of H_2_S in the brain. Emphasizes the protective effect of H_2_S against oxidative stress by enhancing the synthesis of glutathione (GSH) and directly scavenging ROS. Explores the potential therapeutic applications of H_2_S donors in various neurological conditions, including Parkinson’s and Alzheimer’s diseases, offering a more refined understanding of H_2_S’s multifaceted role.	The article elucidates the complex role of hydrogen sulfide (H_2_S) in neuroprotection, highlighting its potential therapeutic applications in neurodegenerative disorders and emphasizing the need for further research to understand its multifaceted functions.
[89]	H_2_S regulation of ferroptosis attenuates sepsis-induced cardiomyopathy	The article presents new insights into the role of sodium hydrosulfide (NaHS) in alleviating sepsis-induced cardiomyopathy (SIC). Specifically, it demonstrates that NaHS mitigates oxidative stress and lipid peroxidation in cardiomyocytes, highlighting its potential as a therapeutic target for SIC. This adds to understanding NaHS’s anti-inflammatory, anti-oxidative stress, and anti-apoptotic properties and its regulation of pathways involved in sepsis multiorgan injury.	The study reveals that NaHS alleviates sepsis-induced cardiomyopathy by reducing oxidative stress and lipid peroxidation, suggesting therapeutic potential.
[90]	Sulfur content in foods and beverages and its role in human and animal metabolism: A scoping review of recent studies	The text highlights recent insights into sulfur dioxide’s physiological and toxicological roles (SO_2_) and its derivatives, emphasizing their complex effects on oxidative stress, gastrointestinal health, and food preservation. It contrasts previous understanding by detailing SO_2_’s potential preventive role in colitis, its impact on the gut microbiome, and its intricate interaction with various biological pathways.	The article reveals new insights into SO_2_’s roles in oxidative stress, colitis prevention, and gut microbiome interaction.
[91]	Dose-Dependent Effect of Hydrogen Sulfide in Cyclophosphamide-Induced Hepatotoxicity in Rats	This study introduces new insights into the role of hydrogen sulfide (H_2_S) in mitigating cyclophosphamide (CP)-induced hepatotoxicity. It emphasizes the protective effects of NaHS, an H_2_S donor, against liver damage caused by CP, a chemotherapeutic agent. The research highlights how H_2_S attenuates oxidative stress in the liver, including regulating critical enzymes and interactions with nitric oxide (NO). This adds to the existing knowledge by elucidating the potential therapeutic applications of H_2_S in preventing drug-induced liver injury.	The study reveals hydrogen sulfide’s potential in mitigating cyclophosphamide-induced hepatotoxicity, highlighting therapeutic applications in preventing drug-induced liver injury.

### 3.2. Hydrogen Sulfide and Cellular Homeostasis—Last-Year Data

H_2_S has garnered considerable attention as an endogenous gaseous signaling molecule that is a critical regulator of cellular homeostasis. Cell homeostasis refers to maintaining a stable, constant cell condition governed by a complex network of regulatory mechanisms [92,93]. H_2_S, acting as a gasotransmitter, has been increasingly involved in numerous physiological processes integral to maintaining cellular homeostasis [10,94].

To begin with, the role of H_2_S in cellular bioenergetics is significant. It is widely known that the cell’s primary powerhouse, the mitochondria, governs energy production via oxidative phosphorylation, producing ATP, the cell’s energy currency. H_2_S has been shown to modulate mitochondrial function at several levels. One of the ways it does this is by influencing the function of cytochrome c oxidase, an element of the mitochondrial electron transport chain, thereby affecting ATP production. By acting on the mitochondria, H_2_S regulates reactive oxygen species (ROS) production, linking its role to oxidative stress and cellular redox homeostasis [11,95,96].

The role of H_2_S in cellular homeostasis extends to metabolic regulation. H_2_S has been connected with glucose homeostasis, contributing to insulin secretion and sensitivity. It modulates the activity of several enzymes involved in the citric acid cycle, glycolysis, and gluconeogenesis, thereby influencing overall energy metabolism. It can enhance glucose uptake in peripheral tissues and has been involved in lipid metabolism, linking its role to metabolic disorders [97].

H_2_S also plays a pivotal role in regulating cellular signal transduction. It can modulate the activity of a wide range of proteins through a process known as persulfidation, wherein a sulfur atom is added to the protein, affecting its function. This post-translational modification can impact various signaling pathways and has been linked to autophagy, inflammation, and cellular stress response [13,83].

The role of H_2_S in cellular proliferation and apoptosis further exemplifies its importance in cellular homeostasis. It can inhibit apoptosis and promote cell survival at physiological concentrations, while at higher concentrations, it can promote apoptosis. The dichotomy in the effects of H_2_S on cell survival and apoptosis is critical to processes, such as tissue repair and regeneration and the response to cellular stress. H_2_S achieves this through several mechanisms, including the modulation of mitochondrial function, the regulation of apoptotic proteins, such as Bcl-2 and Bax, and the modulation of caspase activity [63,94,98].

H_2_S has also emerged as a critical regulator in cellular homeostasis through epigenetic mechanisms. Through its role in modulating transcription factors, chromatin remodeling, and methylation patterns, H_2_S intricately controls cellular functions [99,100].

Given its involvement in these diverse cellular processes, it is unsurprising that dysregulation of H_2_S production or metabolism has been linked to various pathological conditions. A decrease in H_2_S levels has been reported in several diseases, including cardiovascular disease, neurodegenerative disorders, and kidney disease [101]. On the other hand, excessive production of H_2_S has been linked to conditions, such as Down syndrome [102] and cancer [103,104], underscoring the importance of a delicate balance in H_2_S homeostasis and also the role of H2S in general cellular homeostasis (Table 2).

**Table 2:** Synthetic Data on Hydrogen Sulfide and Cellular Homeostasis.

Ref.	Title	Synthetic Data on Hydrogen Sulfide and Cellular Homeostasis	Outcomes
[11]	Hydrogen Sulfide (H_2_S) Signaling as a Protective Mechanism against Endogenous and Exogenous Neurotoxicants	H_2_S mediates redox homeostasis, inflammatory response, mitochondrial functions, and synaptic transmission. New findings on H_2_S’s regulation of SIRT1 expression, its interaction with CREB, and its involvement in reducing homocysteine (Hcy)-induced endoplasmic reticulum stress (ERS), thus extending previous knowledge on its neuroprotective effects.	New insights detail hydrogen sulfide’s role in neuroprotection, redox homeostasis, SIRT1 regulation, and the reduction in Hcy-induced ERS.
[13]	From Gasotransmitter to Immunomodulator: The Emerging Role of Hydrogen Sulfide in Macrophage Biology	The paper emphasizes H_2_S as a potent inflammatory mediator, modulating macrophage activities, such as migration, phagocytosis, and cytokine production. Additionally, the article underscores H_2_S’s involvement in maintaining GSH levels, thus contributing to cellular redox homeostasis.	Recent findings emphasize H_2_S as an inflammatory mediator in macrophages, contributing to cellular homeostasis.
[63]	Recent Development of the Molecular and Cellular Mechanisms of Hydrogen Sulfide Gasotransmitter	The article highlights novel insights into H_2_S’s role in cellular homeostasis, emphasizing the interactions between H_2_S and other gasotransmitters, like NO and CO. It particularly illustrates how H_2_S can persulfidate specific enzymes, impacting various signaling pathways, and reveals new findings on H_2_S in the regulation of endocytosis, autophagy, and renal sodium homeostasis dysfunction. This information expands our understanding of H_2_S’s multifaceted roles in cellular processes.	H_2_S regulates endocytosis, autophagy, and renal sodium homeostasis dysfunction.
[83]	Protein persulfidation: Rewiring the hydrogen sulfide signaling in cell stress response	The article emphasizes the process of persulfidation as a biological switch in the cell stress response. The explanation of H_2_S’s biphasic model, where its levels operate within an optimal concentration range for health, represents an advancement in understanding its dynamic regulation of biological homeostasis, potentially opening novel therapeutic avenues.	Persulfidation’s significance in stress response and the revelation of H_2_S’s biphasic model
[94]	The Impact of Drugs on Hydrogen Sulfide Homeostasis in Mammals	This review highlights a novel aspect of H_2_S’s role in cellular homeostasis by emphasizing its interaction with commonly prescribed pharmacological drugs. Specifically, it catalogs the impact of these drugs on H_2_S production in mammalian cells and tissues, providing new insights into their influence on various physiological and pathological conditions.	The review reveals the impact of pharmacological drugs on hydrogen sulfide production in mammalian systems.
[96]	Mitochondria in endothelial cells angiogenesis and function: current understanding and future perspectives	The text highlights novel insights into H_2_S’s role in endothelial cell (EC) angiogenesis and mitochondrial function. Specifically, it elucidates the dual role of H_2_S in mitochondrial metabolism, showing its function enhancement at low concentrations and inhibition at high concentrations.	Explanation of H_2_S’s impact on specific enzymes and signaling pathways related to angiogenesis.
[97]	H_2_S- and Redox-State-Mediated PTP1B S-Sulfhydration in Insulin Signaling	This article introduces new insights into the role of H_2_S in cellular homeostasis, specifically in the context of insulin signaling. Furthermore, the report reveals the differential effects of insulin and metformin on H_2_S and GSH levels in different cell lines, thereby enriching our understanding of the role of H_2_S in insulin signaling and redox regulation.	It emphasizes the significance of PTP1B S-sulfhydration mediated by H_2_S and the redox state in insulin response and regulation.
[98]	Hydrogen sulfide plays an important role by regulating endoplasmic reticulum stress in myocardial diseases	This article introduces the emerging understanding of H_2_S’s role in regulating endoplasmic reticulum (ER) stress in myocardial diseases, a subject not widely explored until recently. The text emphasizes the novel findings on H_2_S’s involvement in physiological and pathological processes related to ER stress.	H_2_S’s involvement in physiological and pathological processes related to ER stress.
[99]	Epigenetic regulation of macrophage polarization in wound healing	The article explores the intricate role of H_2_S in the polarization and function of macrophages in cardiac pathophysiology, including atherosclerosis. Specifically, it highlights the inhibition of cystathionine γ-lyase (CSE) expression and H_2_S production in macrophages by homocysteine (Hcy) and the contribution of the dysregulated CSE-H_2_S signaling pathway to atherosclerosis pathogenesis.	Dysregulation of the CSE- H_2_S signaling pathway in macrophages contributes to atherosclerosis pathogenesis, with homocysteine (Hcy) affecting macrophage polarization.

### 3.3. H_2_S Therapeutic Potential

H_2_S research has rapidly expanded from a basic biological understanding to clinical and translational science, demonstrating immense therapeutic potential. As elaborated in previous sections, the nuanced roles of H_2_S in oxidative stress and cellular homeostasis make it an attractive molecule for therapeutic targeting, especially in conditions characterized by oxidative stress, inflammation, and metabolic imbalance.

From the cardiovascular perspective, the vasodilatory properties of H_2_S have driven exploration into its potential for treating hypertension and other cardiovascular diseases. Drugs that release H_2_S or enhance endogenous H_2_S production could potentially be employed to ameliorate hypertension and atherosclerosis and reduce the risk of heart attacks. Also, the anti-inflammatory properties of H_2_S suggest the potential to treat various inflammatory conditions, ranging from autoimmune diseases to systemic inflammation seen in sepsis [22].

In neurodegenerative diseases, like Alzheimer’s and Parkinson’s, which involve oxidative stress and neuronal death, H_2_S’s antioxidative and anti-apoptotic properties could be leveraged to slow disease progression. Preclinical studies using H_2_S donors have shown promising results in models of neurodegeneration [105,106].

The capacity of H_2_S to regulate glucose uptake and energy metabolism might be harnessed for managing metabolic diseases, such as diabetes [107,108]. Modulating H_2_S levels could improve glucose homeostasis and insulin sensitivity, potentially serving as an adjunct to existing therapies [44].

Despite the therapeutic potential, the journey from bench to bedside is laden with challenges. First, the concentration-dependent dual effects of H_2_S—protective at physiological levels and toxic at high concentrations—necessitate precise control over therapeutic dosing. Developing methods to precisely modulate H_2_S levels in specific tissues or cell types without systemic side effects remains a significant challenge.

Moreover, while we have a broad understanding of the diverse roles of H_2_S, the underlying molecular and biochemical mechanisms remain less understood. For instance, the post-translational modification known as persulfidation, pivotal in H_2_S biology, needs further exploration. A more profound understanding of these mechanisms is vital for developing targeted therapies.

Controversies exist, too, adding to the complexity. Some studies have reported contradictory results, likely due to differences in experimental models, techniques, and conditions. A consensus on the standard methodologies for measuring H_2_S levels and their effects is urgently required to harmonize these disparities and advance the field.

Looking toward the future, there is a need for more in-depth studies elucidating the interplay between H_2_S, oxidative stress, and cellular homeostasis. Large-scale, well-controlled clinical trials are necessary to assess the safety and efficacy of H_2_S-based therapies. Innovative strategies for targeted H_2_S delivery at desired locations and concentrations will be crucial for practical therapeutic applications.

## 4. Discussion

In this comprehensive one-year review, we have ventured into the intricate roles of hydrogen sulfide (H_2_S) in maintaining cellular homeostasis and orchestrating the regulation of oxidative stress. By synthesizing the recent literature, we have unearthed fresh insights into the multifaceted functions of H_2_S and its profound implications for both physiological equilibrium and pathological perturbations.

Our endeavor bridges the chasm between nascent research and established knowledge surrounding H_2_S, underscoring its dynamic participation in maintaining cellular equilibrium and modulating oxidative stress. While earlier reviews laid essential groundwork, our approach stands apart in several pivotal ways. We meticulously curated and exhaustively analyzed articles published over the past year, thus capturing the latest strides and emerging trends in H_2_S research. This focused timeline facilitated the presentation of cutting-edge discoveries that augment the ongoing discourse on H_2_S’s multifarious roles.

H_2_S has gained unprecedented attention as a signaling molecule [96] in recent years. Its pivotal roles across animals and plants [107,108] in diverse cellular processes have triggered intensive exploration. Divergent as animals and plants may be in terms of physiology, metabolism, and anatomy, their pathways intersect when it comes to hydrogen sulfide’s indispensable roles. Redox signaling, ion channel regulation, and stress response mechanisms, which H_2_S masterfully orchestrates, resonate profoundly in both these biological domains. Furthermore, H_2_S displays therapeutic potential in both realms, as it mitigates cardiovascular disorders in animals and enhances stress tolerance in plants, emphasizing its cross-species therapeutic applicability [11,95,96].

Emerging research spotlights H_2_S’s intricate interaction with lipid peroxidation, a process involving oxidative lipid degradation. As a regulator of lipid peroxidation, H_2_S exhibits both protective and regulatory effects, influencing the expression and activity of enzymes pivotal in lipid peroxidation pathways. Notably, our exploration delves into the connection between H_2_S and ferroptosis—a recently identified form of regulated cell death marked by iron-dependent lipid peroxide accumulation. H_2_S’s multifaceted role in modulating lipid peroxidation and cellular redox balance positions it as a potent inhibitor of ferroptosis. Its capacity to scavenge lipid peroxides and ROS and promote the expression of antioxidant enzymes, such as glutathione peroxidase 4 (GPX4), highlights its regulatory prowess in thwarting ferroptotic processes [10,41].

Our review casts light on H_2_S’s intricate involvement in the aging process. With its multifaceted roles in cellular signaling, antioxidant defense, and metabolic regulation, H_2_S significantly impacts pathways linked to aging [69,70]. By scavenging ROS and inhibiting lipid peroxidation, H_2_S safeguards cellular constituents, such as DNA, proteins, and lipids, from oxidative damage—fundamental to aging-associated oxidative stress mitigation. Moreover, its anti-inflammatory effects contribute to attenuating the inflammatory state often linked to healthier aging [85].

Mitochondrial dysfunction is a hallmark of aging, and here, H_2_S’s protective function shines. It preserves mitochondrial integrity by sustaining electron transport chain activity, reducing ROS production, and fostering mitochondrial biogenesis. These mechanisms collectively bolster cellular energy production and overall health [76].

Proper proteostasis, or protein homeostasis, is paramount for averting the accumulation of misfolded proteins—a hallmark of aging-related ailments. H_2_S plays a pivotal role in maintaining proteostasis through the modulation of heat shock proteins (HSPs) and proteasomal degradation mechanisms. Additionally, H_2_S’s influence on cellular senescence, a state of irreversible growth arrest linked to aging, is evident. By affecting the expression of senescence-associated markers and promoting the clearance of senescent cells through autophagy, H_2_S emerges as a potent modulator of cellular senescence [85].

Our review serves as an impetus for future explorations by pinpointing areas ripe for further investigation. The elucidation of H_2_S’s interactions with antioxidant defense systems, a cellular redox state, and pivotal signaling cascades furnishes a foundational framework for mechanistic dissections. This knowledge frontier beckons researchers to unravel the precise molecular mechanisms governing H_2_S’s protective effects. Furthermore, the interplay between H_2_S and other antioxidants, such as coenzyme Q10, unfurls exciting avenues for collaborative research.

## 5. Conclusions

H_2_S plays a multifaceted role in maintaining cellular homeostasis, impacting cellular bioenergetics, redox balance, signal transduction, cell survival and apoptosis, and metabolic regulation. Despite the significant efforts to understand the role of H_2_S in cellular homeostasis, several questions remain. For instance, how is H_2_S homeostasis maintained at the cellular level? What are the specific targets of H_2_S in the cell? Further exploring these questions could pave the way for developing therapeutic strategies to modulate H_2_S levels to treat various diseases [42].

One of the central threads that emerged from this review is the profound effect of H_2_S on the redox status of cells. H_2_S exhibits antioxidant properties, can induce the production of glutathione, and activates the Nrf2 pathway, all contributing to the regulation of oxidative stress and the maintenance of cellular redox balance [11].

Moreover, the significance of H_2_S in cellular energy metabolism was elucidated. H_2_S interacts with multiple enzymatic components of energy metabolism pathways, regulates glucose uptake, and maintains mitochondrial function, thus playing a critical role in overall energy homeostasis [96].

The review also highlights the controversies and challenges within this rapidly advancing field. Despite extensive studies, the precise mechanisms underlying the biological effects of H_2_S remain to be fully elucidated. Notably, the post-translational modification known as persulfidation, through which H_2_S exerts influence, necessitates further exploration. The delicate balance of H_2_S homeostasis in the cell and the dichotomy of its protective and deleterious effects warrant deeper understanding.

In conclusion, the study of H_2_S represents a frontier in our understanding of gasotransmitters, oxidative stress, and cellular homeostasis. The complexity and breadth of H_2_S influence in biological systems make it an attractive target for future research and therapeutic developments. We hope this review will spark a further investigation into this critical molecule, ultimately paving the way for new therapeutic approaches for various diseases where oxidative stress and disturbances in cellular homeostasis are implicated.
